# Supplementary material for: Context-Dependent Regulation of Peripheral Nerve Abundance by the PI3K Pathway in the Tumor Microenvironment of Head and Neck Squamous Cell Carcinoma
Source: Cells. 2024 Jun 14;13(12):1033. doi: 10.3390/cells13121033 (PMC11202044; doi:10.3390/cells13121033)
Supplement: Supplementary file 1 [file cells-13-01033-s001.zip › Supplementary files/supp/Figure S1_final.pdf]

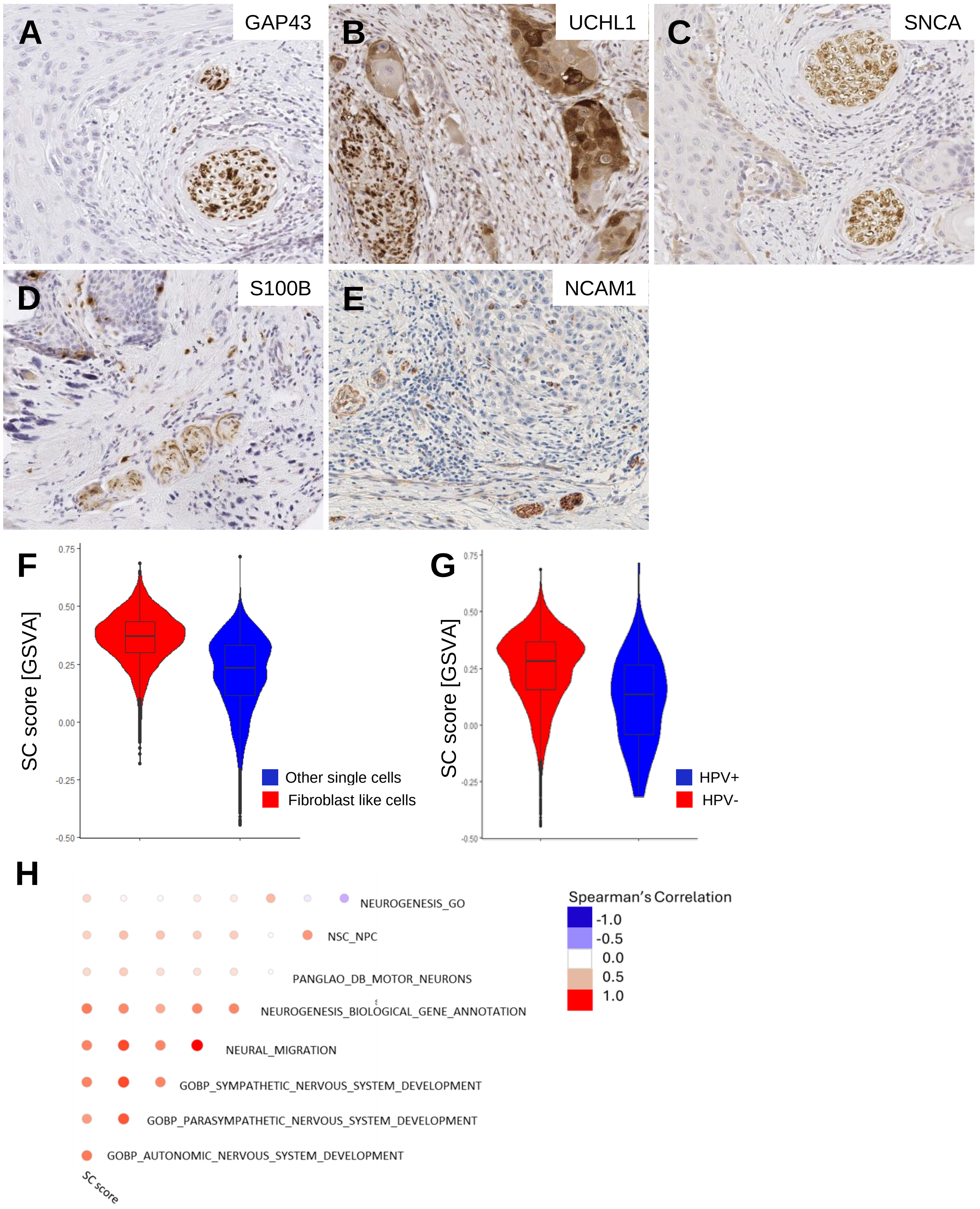

Figure S1: Confirmation of the SC-related 43-gene set by IHC and scRNA-seq analysis. (A-E) Representative images of an IHC staining with FFPE tumor sections and antibodies for indicated proteins showing a positive staining (brown signal) in neuronal structures, malignant epithelial cells and stromal cells of the TME. Hematoxylin staining was used to visualize the tissue architecture. Violin plots illustrate the distribution of SC scores based on scRNA-seq data for fibroblast-like versus other cells (F), and single cells from HPV-negative versus HPV-positive tumors (G). Spearman's correlation coefficient matrix for GSVA scores of the SC-related 43-gene set and indicated gene sets related to specific neural gene sets based on bulk RNA-seq data from TCGA-HNSCC (H).
