# Supplementary material for: Context-Dependent Regulation of Peripheral Nerve Abundance by the PI3K Pathway in the Tumor Microenvironment of Head and Neck Squamous Cell Carcinoma
Source: Cells. 2024 Jun 14;13(12):1033. doi: 10.3390/cells13121033 (PMC11202044; doi:10.3390/cells13121033)
Supplement: Supplementary file 1 [file cells-13-01033-s001.zip › Supplementary files/supp/Figure S2_final.pdf]

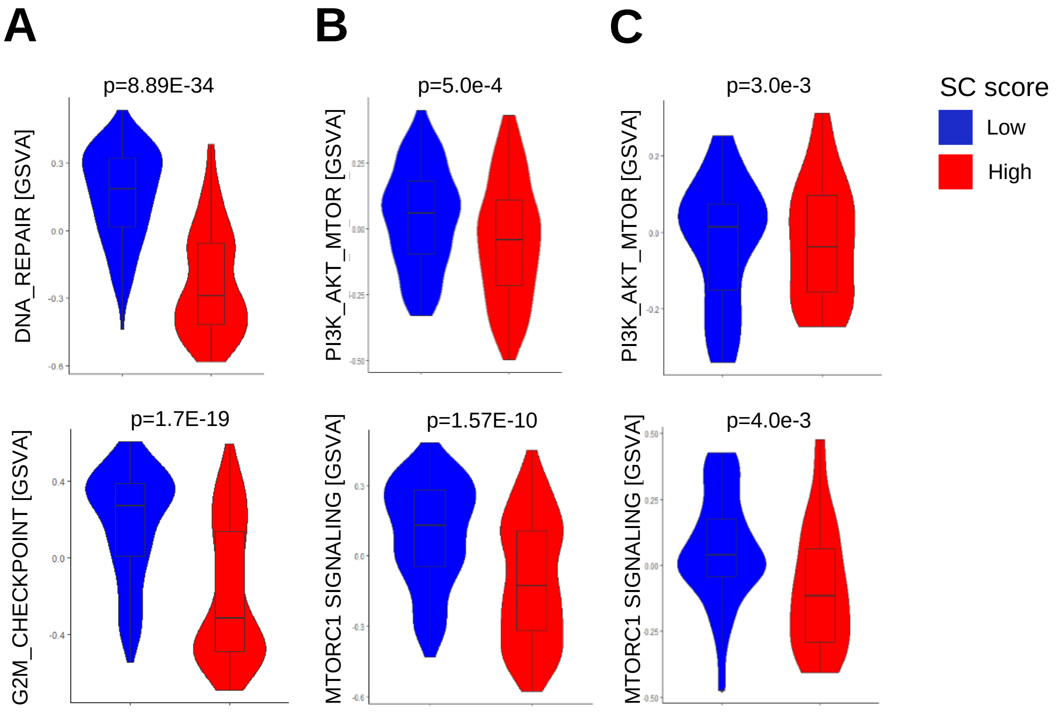

Figure S2: GSVA analysis of hallmark gene sets from MSigSB for TCGA-HNSC and CPTAC-HNSC. Violin plots showing differences in the GSVA scores for indicated hallmark gene sets from MSigDB for tumors with a low versus high SC scores from TCGA-HNSC (A), the subset of HPV16-negative tumors from TCGA-HNSC (B), and tumors from CPTAC-HNSC (C).
