# Supplementary material for: Context-Dependent Regulation of Peripheral Nerve Abundance by the PI3K Pathway in the Tumor Microenvironment of Head and Neck Squamous Cell Carcinoma
Source: Cells. 2024 Jun 14;13(12):1033. doi: 10.3390/cells13121033 (PMC11202044; doi:10.3390/cells13121033)
Supplement: Supplementary file 1 [file cells-13-01033-s001.zip › Supplementary files/supp/Figure S3_final.pdf]

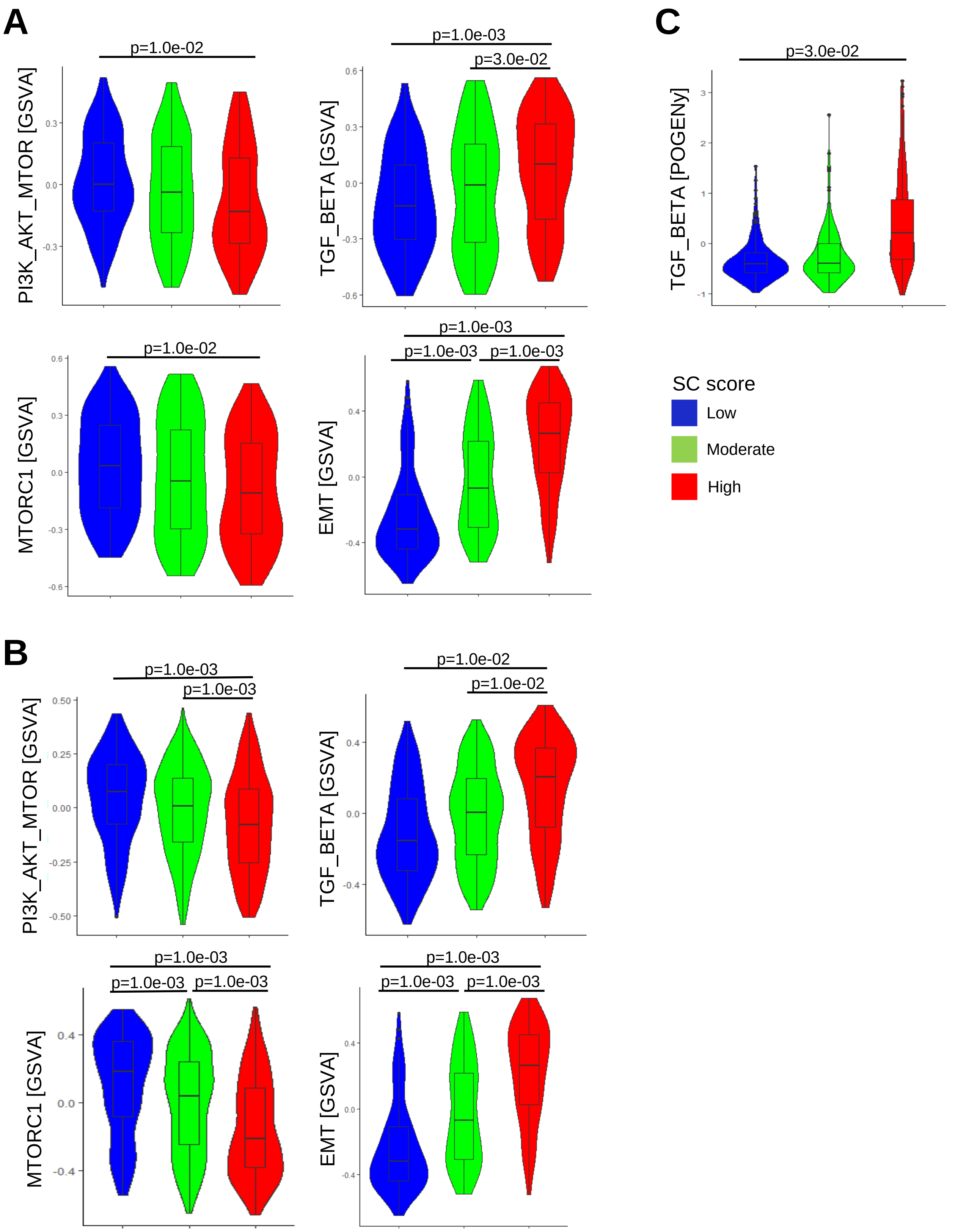


Figure S3:GSVA analysis of hallmark gene sets from MSigSB for TCGA-CESC and TCGA-BRCA and PROGENy analysis for TCGA-HNSC. Violin plots showing differences in the GSVA scores for indicated hallmark gene sets from MSigDB for tumors from TCGA-CESC (A) and TCGA-BRCA (B). (C) Violin plot showing PROGENy scores for TGFβ signaling of TCGA-HNSC tumors.
