# Supplementary material for: Context-Dependent Regulation of Peripheral Nerve Abundance by the PI3K Pathway in the Tumor Microenvironment of Head and Neck Squamous Cell Carcinoma
Source: Cells. 2024 Jun 14;13(12):1033. doi: 10.3390/cells13121033 (PMC11202044; doi:10.3390/cells13121033)
Supplement: Supplementary file 1 [file cells-13-01033-s001.zip › Supplementary files/supp/Figure S4_final.pdf]

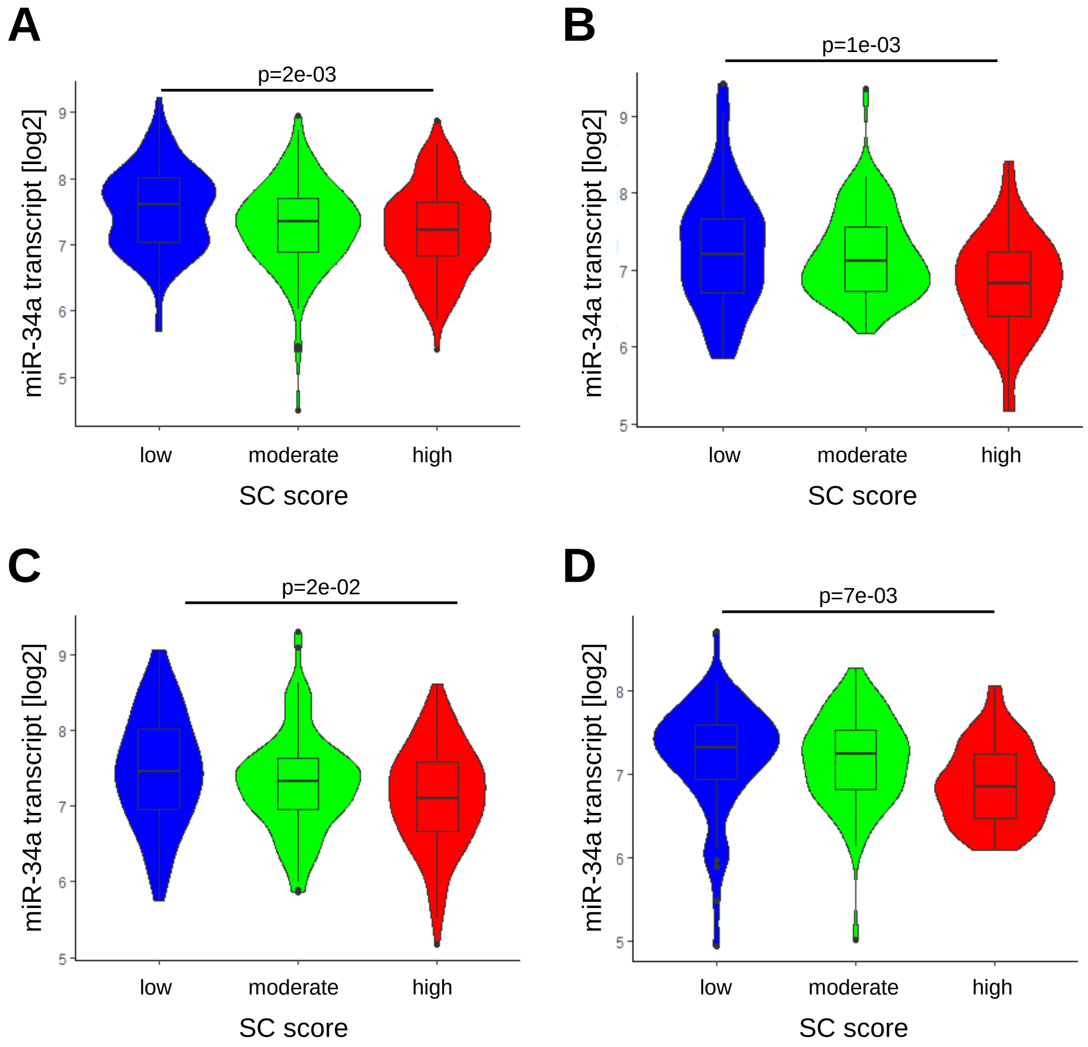

Figure S4: MiR-34a expression in other tumors from TCGA. Violin plots showing miR-34a transcript levels for tumors with low, moderate and high SC scores from TCGA-LUSC (A), TCGA-ESCA (B), TCGA-ESCA (C) and TCGA-PAAD (D).
