# Supplementary material for: Context-Dependent Regulation of Peripheral Nerve Abundance by the PI3K Pathway in the Tumor Microenvironment of Head and Neck Squamous Cell Carcinoma
Source: Cells. 2024 Jun 14;13(12):1033. doi: 10.3390/cells13121033 (PMC11202044; doi:10.3390/cells13121033)
Supplement: Supplementary file 1 [file cells-13-01033-s001.zip › Supplementary files/supp/Figure S5_final.pdf]

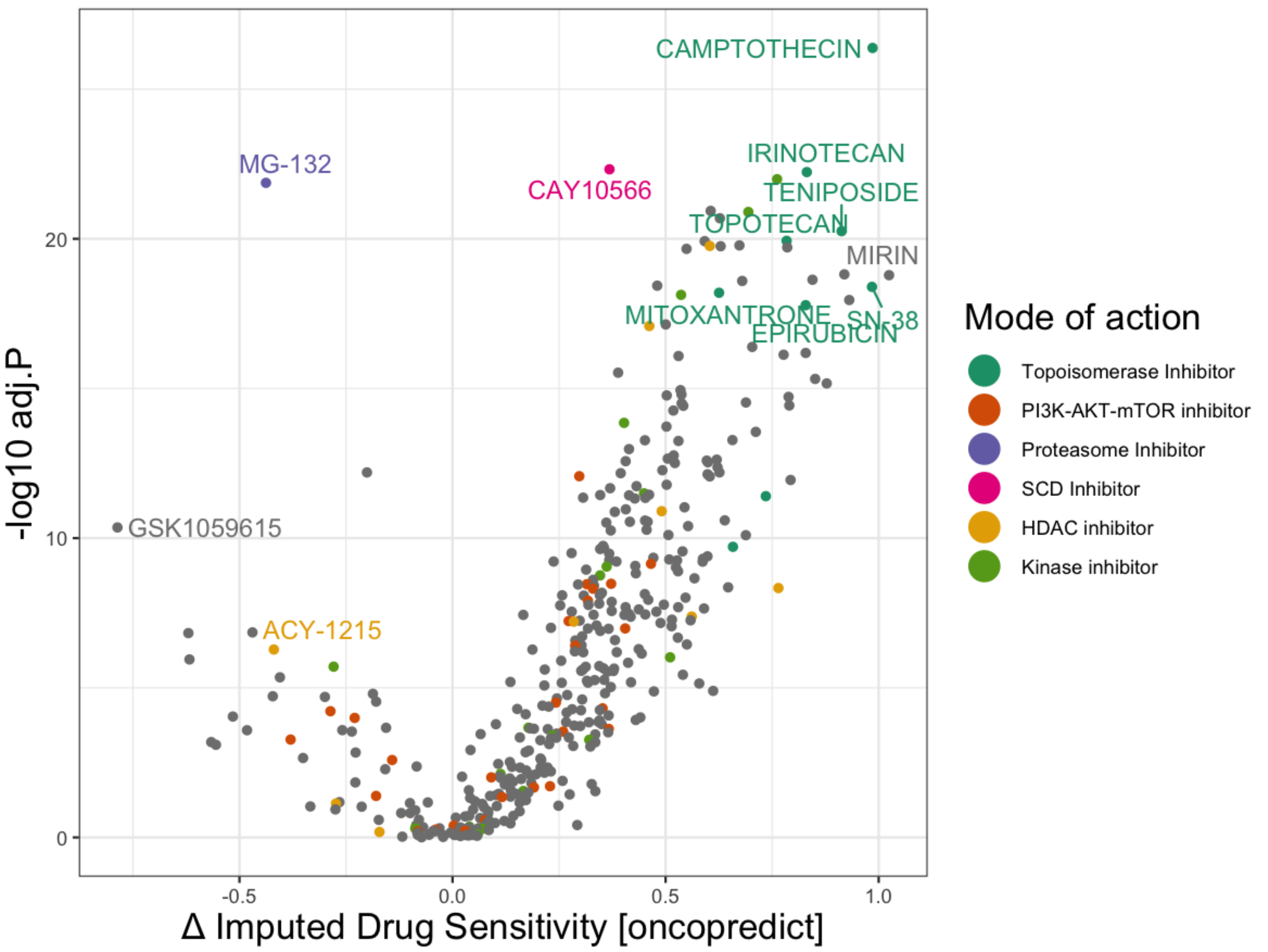

Figure S5: In-silico drug screening for TCGA-HNSC. Dot plot showing the differences in drug sensitivity scores imputed by Oncopredict for tumors with a low versus high SC scores from TCGA-HNSC. Selected drugs with a similar target profile are indicated and highlighted by the color code.
